# Mutational and copy number asset of primary sporadic neuroendocrine tumors of the small intestine

**DOI:** 10.1007/s00428-018-2450-x

**Published:** 2018-09-16

**Authors:** Michele Simbolo, Caterina Vicentini, Andrea Mafficini, Matteo Fassan, Serena Pedron, Vincenzo Corbo, Luca Mastracci, Borislav Rusev, Corrado Pedrazzani, Luca Landoni, Federica Grillo, Sara Cingarlini, Guido Rindi, Claudio Luchini, Aldo Scarpa, Rita T. Lawlor

**Affiliations:** 1 ARC-Net Research Centre, University and Hospital Trust of Verona, Policlinico GB Rossi, Piazzale L.A. Scuro, 10, Piastra Odontoiatrica (II floor), Verona, Italy; 2 Department of Diagnostics and Public Health, Section of Pathology, University and Hospital Trust of Verona, Verona, Italy; 3 Present Address: Department of Medicine (DIMED), Surgical Pathology and Cytopathology Unit, University of Padua, Padua, Italy; 4 Department of Surgical and Diagnostic Sciences (DISC), University of Genoa and IRCCS S. Martino-IST University Hospital, Genoa, Italy; 5 Department of Surgery, General and Hepatobiliary Surgery, University and Hospital Trust of Verona, Verona, Italy; 6 Department of Surgery and Oncology, Unit of Surgery B, The Pancreas Institute, University and Hospital Trust of Verona, Verona, Italy; 7 Department of Medicine, Section of Medical Oncology, University and Hospital Trust of Verona, Verona, Italy; 8 Intitute of Pathology, Università Cattolica-IRCCS Fondazione Policlinico A. Gemelli, Rome, Italy

**Keywords:** Neuroendocrine tumors, Small intestine, *SRC*, Prognostic markers

## Abstract

Small intestine neuroendocrine tumors (SI-NETs) represent the most common histotype among small intestine neoplasms, and metastatic disease is usually present at diagnosis. A retrospective series of 52 sporadic primary surgically resected SI-NETs, which were metastatic at diagnosis, was analyzed by high-coverage target sequencing (HCTS) for the mutational status of 57 genes and copy number status of 40 genes selected from recently published genome sequencing data. Seven genes were found to be recurrently mutated: *CDKN1B* (9.6%), *APC* and *CDKN2C* (each 7.7%), *BRAF*, *KRAS*, *PIK3CA*, and *TP53* (each 3.8%). Copy number analysis showed frequent allelic loss of 4 genes located on chromosome 18 (*BCL2*, *CDH19*, *DCC*, and *SMAD4*) in 23/52 (44.2%) and losses on chromosomes 11 (38%) and 16 (15%). Other recurrent copy number variations were gains for genes located on chromosomes 4 (31%), 5 (27%), 14 (36%), and 20 (20%). Univariate survival analysis showed that *SRC* gene copy number gains were associated with a poorer prognosis (*p* = 0.047). Recurrent copy number variations are important events in SI-NET and *SRC* may represent a novel prognostic biomarker for this tumor type.

**Electronic supplementary material:**

The online version of this article (10.1007/s00428-018-2450-x) contains supplementary material, which is available to authorized users.

## Introduction

Small intestinal neuroendocrine tumor (SI-NET) is the most frequent cancer type of the small bowel [21]. Despite slow-growing [18, 19], SI-NET is a deadly disease usually diagnosed at metastatic stage [12]. Many patients are asymptomatic, and the disease is often diagnosed incidentally. Less frequently SI-NETs are responsible for a carcinoid syndrome, which usually occurs in patients with liver metastases, due to excessive production and release of hormones and substances such as serotonin and prostaglandins [5].

The genetics of SI-NET remains elusive. Genome sequencing of the two independent series so far published provided dissonant information regarding recurrent somatic gene mutations, suggesting multigene potential mechanisms of carcinogenesis [2, 10]. In particular, a combination of whole-genome and exome sequencing performed on 50 SI-NETs showed somatic mutations of the *CDKN1B* gene in 14% of cases [10], which were not found in the exome sequencing study of 48 SI-NET [2]. This latter study identified mutations in several other cancer genes, although none was recurrently altered [2]. A single study reported a relatively high frequency of *APC* gene alterations (7 of 30 cases; 23%) [4], but only one mutation was found in one [10] of the two genome sequencing studies [2, 10].

At variance with gene mutations, chromosomal and gene copy number alterations characterize a significant portion of SI-NETs [2, 10, 13]. Consistent chromosomal and gene copy number alterations reported by the same two genome sequencing studies included loss of chromosome 18 in about 50% and of chromosome 16 in up to 18% of cases, whereas gains were in chromosomes 4, 5, 7, 14, and 20 occurring in a range from 10 to 30% of cases [2, 10]. Allelic loss of chromosome 18 is the most frequent anomaly that has been consistently reported in several different studies [1, 7, 11, 14, 15, 24], whereas gains of chromosome 14 [1] and 20pter-p11.21 [11] have been associated with shorter patients’ survival.

In this paper, we studied a well-characterized cohort of 52 primary SI-NETs that were metastatic at diagnosis performing: (i) a targeted deep sequencing mutational analysis for 57 relevant genes; (ii) copy number variation (CNV) analysis of a selected number of 40 genes located in the most frequently altered chromosomes; (iii) survival analysis to investigate the potential clinical relevance of the results.

## Materials and methods

### Patients and samples

A retrospective series (1997–2012) of 52 sporadic surgically resected primary intestinal neuroendocrine tumors (47 ileal, 4 duodenal, and 1 jejunal) were retrieved from the formalin-fixed paraffin-embedded (FFPE) archives of the ARC-Net Biobank at Verona University Hospital and from the University of Genoa/IRCCS S. Martino-IST University Hospital. All cases were reclassified according to WHO 2010 [3] and staging was assessed according to AJCC/UICC 7th edition [9]. Matched normal small intestine samples were used to determine the somatic/germline nature of mutations.

In 25 cases, sufficient material was available for the construction of 1-mm core tissue microarrays (TMAs) using the Galileo CK3500 Arrayer (www.isenet.it), a semiautomatic and computer-assisted TMA platform. Three tissue cores per case were analyzed.

Clinico-pathological characteristics of the sample cohort along with the molecular analyses conducted in this study are reported in Table 1 and Supplementary Table 1.

**Table 1:** Clinical-pathological features of the 52 SI-NETs considered in the study

Clinico-pathological features		Total (52)	[%]
Sex		30M 22F	
Age		46.1 ± 24.0	
Dimension (cm)		2.2 ± 1.3	
Differentiation	WD	49	[94.2]
	PD	3	[5.8]
Grade	G1	36	[69.2]
	G2	13	[25.0]
	G3	3	[5.8]
Stage UICC/ENETS	IIIB	22	[42.3]
	IV	30	[57.7]
R	R1	4	[7.7]
	R0	48	[92.3]
Vascular invasion	Present	35	[67.3]
	Absent	17	[32.7]
Perineural invasion	Present	32	[61.5]
	Absent	20	[38.5]
Necrosis	Present	3	[5.8]
	Absent	49	[94.2]

*WD* well differentiated, *PD* poorly differentiated, *R1* positive resection margins, *R0* negative resection margins

### DNA extraction and qualification

DNA was obtained from matched tumor and normal FFPE tissues, after enrichment for neoplastic cellularity to at least 70% using manual microdissection of 10 consecutive 4-μm sections. DNA was purified using the QIAamp DNA FFPE Tissue Kit (Qiagen) and qualified as reported [25, 27]. Briefly, DNA was quantified using Qubit DNA HS Assay kit (ThermoFisher) and purity was evaluated using NanoDrop ND-2000 [25]; integrity was investigated using the BIOMED protocol, and only DNA samples producing fragments of at least 200 bp were deemed usable for NGS [27].

### RNA extraction and qualification

RNA was obtained from 10 consecutive 6-μm FFPE sections using RecoverAll total nucleic acid isolation kit protocol (ThermoFisher). RNA was quantified using Qubit RNA BR Assay Kit (ThermoFisher) and qualified using Agilent RNA 6000 Nano Kit (Agilent Technologies). A RNA Integrity Number (RIN) over 5 was considered suitable.

### High-coverage target sequencing

Matched tumor/normal DNA from all FFPE samples was subjected to targeted next-generation sequencing (NGS). Three multigene panels were used: the 50-gene Ion AmpliSeq Cancer Hotspot panel v2 (ThermoFisher) and two AmpliSeq custom panels.

The first panel explores mutation status of selected hot-spot regions of 50 cancer-related genes: *ABL1*, *AKT1*, *ALK*, *APC*, *ATM*, *BRAF*, *CDH1*, *CDKN2A*, *CSF1R*, *CTNNB1*, *EGFR*, *ERBB2*, *ERBB4*, *EZH2*, *FBXW7*, *FGFR1*, *FGFR2*, *FGFR3*, *FLT3*, *GNA11*, *GNAS*, *GNAQ*, *HNF1A*, *HRAS*, *IDH1*, *IDH2*, *JAK2*, *JAK3*, *KDR/VEGFR2*, *KIT*, *KRAS*, *MET*, *MLH1*, *MPL*, *NOTCH1*, *NPM1*, *NRAS*, *PDGFRA*, *PIK3CA*, *PTEN*, *PTPN11*, *RB1*, *RET*, *SMAD4*, *SMARCB1*, *SMO*, *SRC*, *STK11*, *TP53*, and *VHL*. Details on target regions of the commercial panel are at http://www.thermofisher.com.

The second panel has been designed for the mutational analysis of 7 genes selected upon the results of published whole genome, exome, and targeted sequencing of SI-NETs series [2, 4, 10, 13]: *ATRX*, *CDKN1B*, *CDKN2C*, *DAXX*, *H3F3A*, *MEN1*, and *TERT.*

The third panel investigates copy number variation (CNV) status of 40 genes reported as altered in SI-NETs [2, 7, 10, 11, 13, 15]: *AKT1*, *APC*, *AURKA*, *BCL2*, *BCL2L2*, *BRAF*, *CDH1*, *CDH19*, *CDKN1B*, *CDKN2A*, *DCC*, *EGFR*, *ERBB2*, *FBXW7*, *FGFR3*, *FHIT*, *FOS*, *GNAS*, *HRAS*, *KDR*, *KIT*, *MAP2K2*, *MDM2*, *MEN1*, *MET*, *MYC*, *MYCL1*, *PDGFRA*, *PIK3CA*, *PIK3CD*, *PTEN*, *RICTOR*, *SDHA*, *SDHB*, *SDHD*, *SMAD4*, *SMAD5*, *SOX12*, *SRC*, and *TP53.* Details on target regions of the CNV custom panel are in Supplementary Table 2A.

Twenty nanograms of DNA were used for each multiplex PCR amplification. The quality of the obtained libraries was evaluated by the Agilent 2100 Bioanalyzer on-chip electrophoresis (Agilent Technologies). Emulsion PCR to construct the libraries of clonal sequences was performed with the Ion OneTouch™ OT2 System (ThermoFisher). Sequencing was run on the Ion Proton (PI, ThermoFisher) loaded with Ion PI Chip v2.

### Sequencing data analysis

Base calling, alignment to the hg19 human reference genome, and variant calling were done using the Torrent Suite Software v.5.0 (ThermoFisher). Called variants were annotated using a custom pipeline based on vcflib (https://github.com/ekg/vcflib), SnpSift [6], the Variant Effect Predictor (VEP) software [17], and NCBI RefSeq database. Filtering of variants was performed by: (i) removal of germline variants and (ii) visual verification of alignments on the IGV software v2.3 [26]. This latter step is key to remove false calls due to technique-dependent mispriming or sample age-related deamination, which cannot be ruled out by automated variant calling and filtering procedures (Supplementary Table 2B).

### CNV analysis using next-generation sequencing

For all samples, a CNV baseline for AmpliSeq custom panels was performed using 10 genomic male DNA extracted from normal tissues included in FFPE samples. CNV was evaluated comparing BAM files of sequenced libraries to baseline through a custom workflow pipeline created on IonReporter 5.0 software. Copy number variation calling of genes included in the Ion AmpliSeq Cancer Hotspot panel v2 and custom panel was performed using followed criteria: (i) a median of the absolute values of all pairwise differences (MAPD) score under 1; (ii) a CNV confidence number major than 20; (iii) a tiles number major than 10. An orthogonal cross-validation using FISH or qPCR was performed. A chromosome integrity number score was evaluated for each sample dividing length of altered chromosomes to length of chromosome regions investigated.

### CNV validation by quantitative-PCR

Q-PCR analysis of copy numbers was applied to all 52 SI-NETs for selected loci. All target and reference assays were purchased from ThermoFisher Scientific. *RNaseP* was used as endogenous control for normalization of analyzed loci. The following assays were used: *AKT1* (Hs02893205), *BCL2* (Hs01500302), *CDH19* (Hs02826809), *DCC* (Hs02317964), *FHIT* (Hs03491211), *MET* (Hs04951661), *PIK3CD* (Hs04540050), *SMAD4* (Hs06483146), *SOX12* (Hs02822764), *SRC* (Hs07169853), *SDHB* (Hs00124581), and *RNaseP* (part number 4403326). The experimental procedure recommended by the manufacturer (Applied Biosystems) was followed. Twenty nanogram genomic DNA was used in the q-PCR reaction, and a negative control was analyzed in parallel. All q-PCR reactions were run in quadruplicate in a 7900HT qRT-PCR machine (Applied Biosystems) using standard cycling conditions of 10 min at 95 °C, followed by 40 cycles of [95 °C for 15 s and at 60 °C for 1 min]. Pooled normal FFPE DNA was used as calibrator and as reference unbiased genome. Microsatellite markers D18S484, D18S51, and D18S1110 were used to confirm LOH/homozygous deletion, respectively, in *DCC*, *BCL2*, and *SMAD4* locus.

### Fluorescent in situ hybridization analysis

FISH analysis was performed to evaluate CNV status of chromosome 18q, 14q, and *SRC* gene. The LSI IGH/BCL2 dual color, dual fusion translocation probe (Vysis Inc./Abbott) was used for chromosome 18q. The TelVysion 14q probe (Vysis Inc./Abbott) was used to evaluate chromosome 14q status as reported in a previous work [1]. A custom FISH probe was developed to evaluate the status of *SRC* gene (chr20q11.23). The custom FISH probe labeled *SRC* (chr20q11.23) in Spectrum Red and the control locus 20p11.21 in Spectrum Green (Empire Genomics). FISH analysis was performed according to the manufacturer’s protocols.

### Expression analysis for SRC gene

An ampliseq RNA custom panel was built to analyze the expression levels of *SRC* and included a series of genes to normalize them (*ACTB*, *CDH17*, *GC*, *HPRT1*, *KCNJ3*, *KIF12*, *MIA2*, *MUC13*, *RNF213*, *RPRM*, *SOX21*). In brief, 1 μg of RNA was retro-transcribed and submitted for library construction. The quality of the obtained libraries was evaluated by the Agilent 2100 Bioanalyzer on-chip electrophoresis (Agilent Technologies). Emulsion PCR to construct the libraries of clonal sequences was performed with the Ion OneTouch™ OT2 System (Life Technologies). Sequencing was performed on Ion PGM loading 318v2 chip, reads were aligned to reference genome (hg19 *Homo sapiens* RNA Canonical Transcript). Normalization was performed dividing the number of reads obtained for *SRC* transcript by the sum of reads obtained for the other genes and expressed as reads per thousands of reads.

### Immunohistochemistry

Immunohistostaining was performed with Leica Microsystems Bond-Max Autostainer System using the antibodies listed in Supplementary Table 3 on 3-μm FFPE sections from tissue blocks containing representative core samples. Immunolabeling for all antibodies was performed according to manufacturer protocols; normal intestinal tissue was used as positive control. Sections incubated without the primary antibodies served as negative control.

### Statistical analysis

One-way ANOVA, Kruskal-Wallis test, Fisher’s test with Monte Carlo simulation, and Fisher’s exact test were used as appropriate; correction for multiple comparisons was performed according to Benjamini-Hochberg. For comparison of Kaplan-Meier survival curves, Mantel-Cox test was used; for multivariable survival analysis, stepwise Cox proportional hazards regression was used; selection of the best model was performed using the “enter” algorithm. For all the analyses, a *p* value below 0.05 was considered as significant. All analyses were performed using Medcalc for Windows version 15.6 (MedCalc Software, Ostend, Belgium) and R v. 3.2.1; multivariable Cox regression was done with R using survival library v.2.38-2.

## Results

### Clinico-pathological characteristics of the series

Clinico-pathological data are detailed in Table 1 and Supplementary Table 1. Tumor grade was G1 in 36 (69.2%), G2 in 13 (25.0%), and G3 in 3 cases (5.8%). Vascular and perineural invasions were present in 35 (67.3%) and 32 (61.5%) cases, respectively. The ENETS and UICC pathologic stages overlapped and were: stage IIIB in 22 and IV in 30.

### Mutational profiles of small intestinal neuroendocrine tumors

**Fig. 1:**
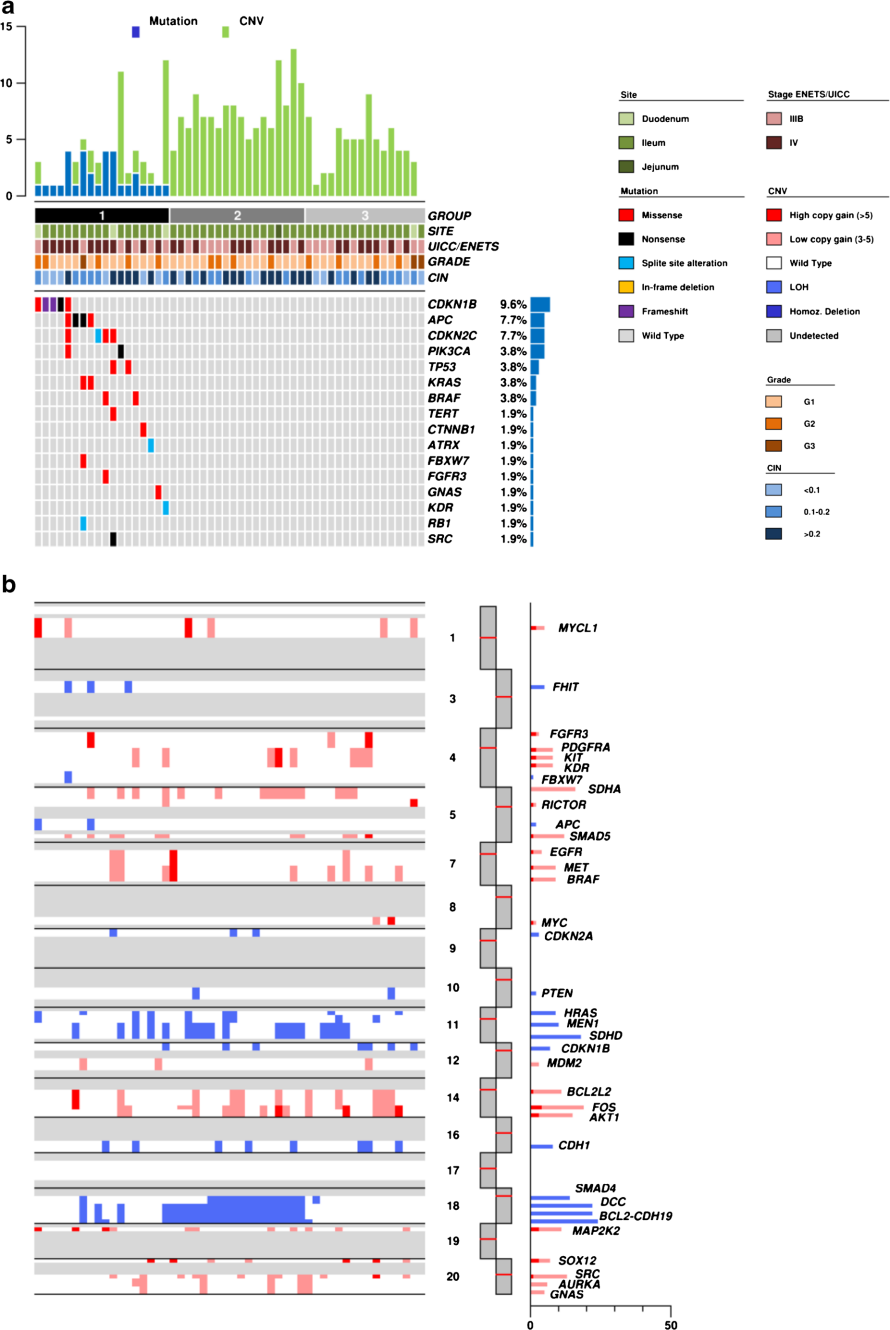
Genetic landscape of 52 SI-NET. **a** The upper histogram shows the number of mutations (blue) and CNV (green) in recurrently altered genes for each sample. The central matrix shows 16 of 57 genes that were mutated in the whole cohort; alterations are annotated by different colors according to their impact on the gene product as illustrated in the panel on the right. **b** Summary of somatic copy number variations determined for 52 human SI-NETs. Significant gains (red) and losses (cyan) were determined for the chromosomal regions and are plotted according to their frequencies on the right

DNA from all samples was successfully amplified in multiplex PCR for the 57 selected genes and adequate libraries for deep sequencing were obtained. The mean read length was 103 base pairs and a mean coverage of 5893× was achieved, with 97.2% target bases covered more than 100×. A minimum coverage of 20× was obtained in all cases. A mean of 0.6 mutation was detected in all series for the 57 genes analyzed. In the whole cohort, 18 (34.6%) samples showed somatic mutations: 12 (23.1%) affected by one mutation; 6 (11.5%) affected by more than one. The remaining 34 (65.4%) tumors resulted wild type for the 57 genes assayed (Fig. 1a, Table 2).

**Table 2:** Mutated genes in the 52 SI-NETs

GENE	Total (52)	[%]	M	N	S	D	F
*APC*	4	[7.7]	1	3	–	–	–
*ATRX*	1	[1.9]	–	–	1	–	–
*BRAF*	2	[3.8]	2	–	–	–	–
*CDKN1B*	5	[9.6]	2	1	–	–	2
*CDKN2C/INK4C*	4	[7.7]	3	–	1	–	–
*CTNNB1*	1	[1.9]	1	–	–	–	–
*FBXW7*	1	[1.9]	1	–	–	–	–
*FGFR3*	1	[1.9]	1	–	–	–	–
*GNAS*	1	[1.9]	1	–	–	–	–
*KRAS*	2	[3.8]	2	–	–	–	–
*PIK3CA*	2	[3.8]	1	1	–	–	–
*RB1*	1	[1.9]	–	–	1	–	–
*SRC*	1	[1.9]	–	1	–	–	–
*TERT*	1	[1.9]	1	–	–	–	–
*TP53*	2	[3.8]	2	–	–	–	–

*M* missense mutation, *N* nonsense mutation, *S* splice site alteration, *D* deletion, *F* frameshift mutation

The most frequently mutated genes were *CDKN1B* (9.6%), followed by *APC* and *CDKN2C* (each 7.7%), *BRAF*, *KRAS*, *PIK3CA*, and *TP53* (each 3.8%). The details of the somatic mutations detected are reported in Supplementary Table 4. Five mutations in tumor suppressor genes (*FGFR3*, *TP53*, *CDKN2C*, *APC*, *RB1*) had a high allelic frequency (> 60%) compatible with homozygous alteration in a large fraction of tumor cells. Fourteen mutations, prevalently in oncogenes or haplo-insufficient tumor suppressor genes, had a frequency between 25 and 57%, compatible with heterozygous alteration in all tumor cells or with homozygous alteration in a consistent (30–50% of total tumor) subpopulation of neoplastic cells. Finally, 11 mutations had a frequency equal to or below 20%, compatible with heterozygous or homozygous alteration of smaller subclones. Notably, the same tumor (e.g., case G5) often bore different mutations with both high and low frequencies, suggesting molecular heterogeneity.

### Copy number variations in selected genes

Copy number variations were evaluated using NGS and validated by q-PCR and FISH. The results are summarized in Table 3, detailed in Supplementary Table 5 and illustrated Fig. 1b and Supplementary Fig. 1.

**Table 3:** Copy number variations for selected genes in 52 SI-NETs. Genes are listed per alphabetical order

Gene	Chromosomal location	Gain or loss	Total (52)	[%]
*AKT1*	14	Gain	16	[30.8]
*APC*	5	LOH	2	[3.8]
*AURKA*	20	Gain	6	[11.5]
*BCL2*	18	LOH	22	[42.3]
*BCL2L2*	14	Gain	11	[21.2]
*BRAF*	7	Gain	9	[17.3]
*CDH1*	16	LOH	8	[15.4]
*CDH19*	18	LOH	24	[46.2]
*CDKN1B*	12	LOH	7	[13.5]
*CDKN2A*	9	LOH	3	[5.8]
*DCC*	18	LOH	22	[42.3]
*EGFR*	7	Gain	4	[7.7]
*ERBB2*	17	Gain	0	[0]
*FBXW7*	4	LOH	1	[1.9]
*FGFR3*	4	Gain	3	[5.8]
*FHIT*	3	LOH	3	[5.8]
*FOS*	14	Gain	19	[36.5]
*GNAS*	20	Gain	5	[9.6]
*HRAS*	11	LOH	9	[17.3]
*KDR*	4	Gain	15	[28.8]
*KIT*	4	Gain	15	[28.8]
*MAP2K2*	19	Gain	11	[21.2]
*MDM2*	12	Gain	3	[5.8]
*MEN1*	11	LOH	7	[13.5]
*MET*	7	Gain	9	[17.3]
*MYC*	8	Gain	2	[3.8]
*MYCL1*	1	Gain	6	[11.5]
*PDGFRA*	4	Gain	15	[28.8]
*PIK3CA*	3	Gain	0	[0]
*PIK3CD*	1	LOH	0	[0]
*PTEN*	10	LOH	2	[3.8]
*RICTOR*	5	Gain	2	[3.8]
*SDHA*	5	Gain	15	[28.8]
*SDHB*	1	LOH	0	[0]
*SDHD*	16	LOH	18	[34.6]
*SMAD4*	18	LOH	15	[28.8]
*SMAD5*	5	Gain	11	[21.2]
*SOX12*	20	Gain	7	[13.5]
*SRC*	20	Gain	13	[25.0]
*TP53*	17	LOH	0	[0]

A mean of 6.5 CNV was found in the series for the 40 genes investigated. Forty-eight (92.3%) samples showed somatic CNV: 1 (1.9%) affected by one CNV and 47 (90.4%) affected by more than one CNV. Four (7.7%) samples showed no CNV event. The most frequent event was loss of a single copy (LOH) of the *CDH19* gene located on 18q22 locus in 24/52 (46.2%). Other genes analyzed and located on chromosome 18q (*BCL2*, *DCC*, *SMAD4*) were mostly affected by LOH. In particular, 23 samples (44.2%) showed copy loss of one or more genes located on this chromosomal region. LOH of *DCC*, *BCL2*, and *SMAD4* were validated using microsatellite marker quantification between tumor and matched normal samples for each specific locus. IHC analysis for CDH1, SMAD4, and CDKN1B was possible for the 25 cases included in the TMAs; in all samples affected by the loss of one gene copy, a significant decrease in the corresponding protein expression was observed (Supplementary Fig. 2). Frequent copy gains were detected in *FOS* gene (19/52, 36.5%) followed by *AKT1* (16/52; 30.8%) and *KIT*, *KDR*, and *PDGFRA* locus (15/52, 28.8%).

### Identification of molecular subgroups

To identify potential molecular subgroups in SI-NET, we separated samples according to their prevalent molecular features. This approach identified three distinct groups as illustrated in Fig. 1. Group 1 included 18 samples characterized by presence of somatic mutations. Groups 2 and 3 together included 34 samples, each with chromosome copy number alterations and no somatic mutations. In particular, group 2 comprised samples featured by LOH in at least 2 genes located on chromosome 18, while group 3 comprised samples with no mutations and low or no LOH affecting chromosome 18 genes.

### Survival analysis

**Fig. 2:**
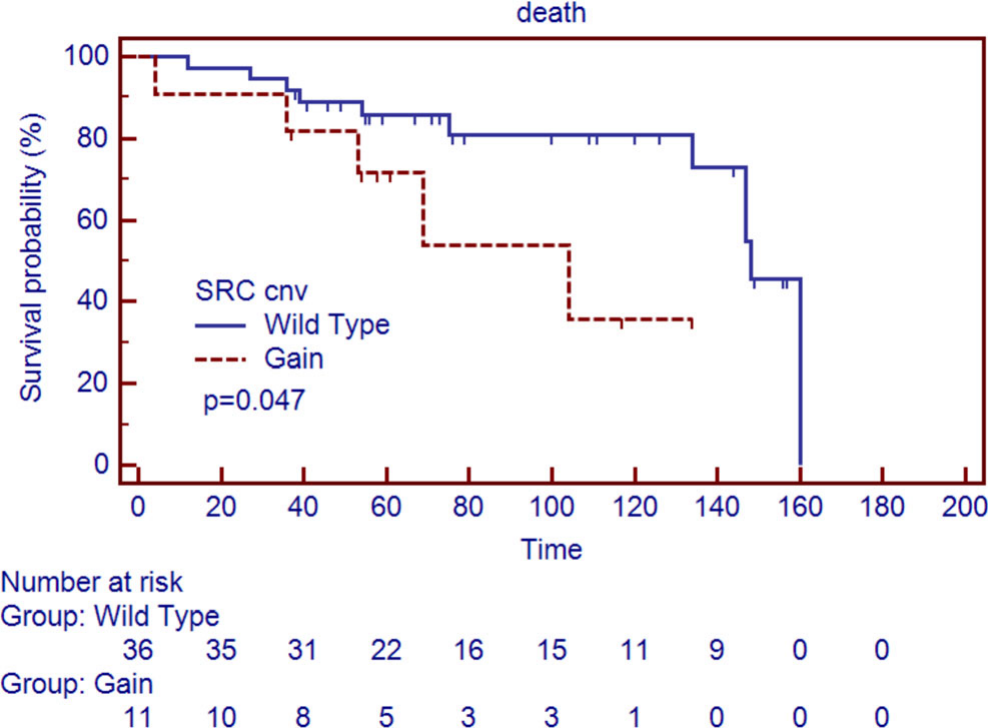
Overall survival according to molecular and pathological features. Overall survival of SI-NETs (*n* = 47) is significantly affected by gain occurring in *SRC locus* gene (*p* = 0.047). Vertical axis indicates percent survival; horizontal axis shows time expressed in months. Kaplan–Meier and log-rank statistics were used to determine levels of significance

Follow-up data was available for 47 cases. Median survival was 71.0 (range 4–160) months and 16 (34.0%) subjects died of disease. To identify clinico-pathological markers of poor prognosis, we considered at univariate analysis: sex, age at diagnosis (under/over 50 years), and presence of nodal or distant metastases (stage IIIB vs. IV). The analysis showed no significant differences about prognosis performance among these markers. When matching clinical data and molecular features, copy gain of *SRC* was identified as the only molecular marker associated to poor prognosis (Fig. 2). Of interest, cases with *SRC* copy gain showed less perineural invasion (*p* = 0.019); no other correlation with clinicopathological features was found. Of note, a strong correlation between age at diagnosis over 50 years and copy gain at the *FOS* gene locus was observed (*p* = 0.001). Molecular subgroups as previously defined by Karpathakis et al. [13] did not associate with any difference in survival (*p* = 0.73).

### SRC expression analysis

To verify the impact of *SRC* copy gain on transcript levels, we performed targeted RNA sequencing on the 14 cases with available residual tissue for research purposes. These included 4 cases with *SRC* copy gains and 10 without *locus* alteration according to CNV analysis. The number of reads obtained for *SRC* was normalized to the sum of the reads obtained for a set of 11 control genes and expressed as normalized counts (reads per thousands of control reads). Cases were grouped according to their genomic *SRC* status (gain vs. wild-type) and Mann-Whitney test was performed to compare them (Supplementary Fig. 3). Normalized counts of SRC transcript were almost 3 times higher in the “Gain” group (median 38.4, 1st–3rd quartiles 31.0–82.2) than in the “Wild-type” group (median 12.6, 1st–3rd quartiles 3.4–19.0, *p* = 0.0020).

## Discussion

The SI-NETs molecular landscape and its clinical implications are still to be fully elucidated.

In this paper, we focused on the major genetic SI-NET alterations in a cohort of 52 sporadic surgically resected advanced SI-NETs. Our study: (i) demonstrated that mutations in gene coding sequences are a relatively rare event; (ii) CNV is relatively frequent, affecting LOH of chromosome 18q in more than 40% of the samples; (iii) *SRC* gene gains are associated to a poorer patients’ survival.

Only 18 (34.6%) samples were characterized by somatic mutations in the 57 analyzed genes in the NGS mutational analysis. Our findings were concordant with those observed by Francis et al. [10] regarding mutation frequencies for *CDKN1B* (9.6%) and *BRAF* (3.8%) genes. Moreover, we found a significant mutational rate for *APC* (7.7%), confirming previous reports obtained by conventional sequencing methods [4, 23].

Unreported findings [4, 10, 22] were the mutations observed in *PIK3CA* (3.8%), *TP53* (3.8%), *KRAS* (3.8%), and *CDKN2C* (7.7%) genes. Cases affected by these mutations did not show any peculiar clinico-pathological feature.

Unlike the poor mutational landscape observed in our series, copy number analysis in selected genes showed recurrent and frequent alterations in the whole cohort. As previously reported in several studies [1, 8, 11, 15, 20], in our series, we also observed copy loss of genes located on chromosome 18 (*DCC*, *SMAD4*, *BCL2*, and *CDH19*; 24/52, 46.2%). Again in line with previous studies [1, 2, 10, 11, 14–16], we identified copy number gains in genes located in chromosomes 4 (*FGFR3*, *KDR*, *KIT*, and *PDGFRA*), 5 (*RICTOR*, *SDHA* and *SMAD5*), 7 (*EGFR* and *BRAF*), 14 (*AKT1*, *BCL2L2*, and *FOS*), and 20 (*AURKA*, *GNAS*, *SOX12*, and *SRC*).

Among the principal genes affected by copy number variations, no mutually exclusive gene was observed between those located on chromosomes 14 and 18. This was at variance from what previously described by Hashemi et al. [11] but similar to what reported by Anderson et al. [1].

Abundance of genes affected by CNV suggests that aberrations in the genomic/chromosomal structure may play an important role in SI-NET biology. Also, survival analysis supported this hypothesis. Correlating clinical data and molecular features, we identified no correlation between prognosis and gene mutation, but did observe a significant relationship by univariate analysis between copy gain of *SRC* gene and poor patient prognosis (*p* = 0.047). Copy gains of the *SRC* gene in SI-NET were first identified by Banck et al. in a series of 48 cases, suggesting *SRC* as potential therapeutic target for this disease [2]. We also report that copy gain of the *SRC* gene is associated with elevated mRNA levels. This was also true for case #V22, presenting 5 copies of *SRC* and an apparently contradictive nonsense mutation. In this case, the relative abundance of the *SRC* transcript was one of the highest (45.8 normalized counts vs. a median of 12.6 normalized counts for *SRC* wild-type cases). Therefore, the nonsense mutation was likely a passenger event with no evident impact on SRC expression.

A recent integrative molecular analysis on 47 SI-NET by Karpathakis et al. [13] highlighted the presence of 3 molecular subgroups via matched copy number and methylation profile results. The most relevant included 26 samples (55% of whole cohort) characterized by loss of chromosome 18 with concurrent CDKN1B mutation and better survival compared to the other two molecular subgroups. In our study, no concurrence of chromosome 18 loss (44.2%) and CDKN1B mutation (9.6%) was observed. Moreover, classifying our cohort into the three groups of Karpathakis et al., we did not observe any statistically significant correlation with prognosis.

In our study, at variance with the current literature [1, 11], poor prognosis was not associated with gains of genes at 20p or 14q loci. To test CNV status of these loci, we analyzed *SOX12* (locus 20p13), *FOS* (locus 14q24.3), and *AKT1* (locus 14q32.33) genes as surrogate for such chromosome arms. Interestingly, *FOS* copy gain was strongly associated with another marker of poor prognosis in our study: advanced age at diagnosis (Fisher’s exact test *p* = 0.001). This might explain why Anderson et al. identified gain of 14q as a poor prognostic marker. In fact, in that study, all samples characterized by this alteration had an advanced age at diagnosis (i.e., over 50 years old) [1].

In conclusion, our study provides additional data to define the genetic landscape of sporadic SI-NETs, highlighting copy number variations as a central molecular event in this tumor type, not only for understanding SI-NET biology, but also with prognostic significance.

## Electronic supplementary material

Supplementary Figure 1 FISH images of representative CNV alterations in SI-NET samples. **A)** Diploid *BCL2* status; **B)** Monosomy of *BCL2*; **C)** Diploid Tel14q status; **D)** Polysomy of Tel14q; **E)** Diploid *SRC* status; **F)** Polysomy of *SRC*. (PNG 2047 kb)

High Resolution Image (TIF 11912 kb)

Supplementary Figure 2 Immunohistochemical validation of the expression of E-cadherin (CDH1), p27 (CDKN1B) and smad4 (SMAD4) proteins in mutated and wild type tumors. Representative immunohistochemical staining of the products of the *CDH1* (E-cadherin), *CDKN1B* (p27) and *SMAD4* (smad4) genes in wild type tumors (left panels) and tumors characterized by LOH of the corresponding genes (right panels). The wild type tumors retained a normal protein expression, which was lost in LOH cases. (PNG 5738 kb)

High Resolution Image (TIF 24929 kb)

Supplementary Figure 3 Small intestinal neuroendocrine tumors with copy gain of *SRC locus* display enhanced levels of SRC mRNA. Normalized expression reads of 14 samples grouped according *SRC* copy number status in Gain (4 samples) and Wild-Type (10 samples). (PNG 104 kb)

High Resolution Image (TIF 5078 kb)

ESM 1 (XLSX 117 kb)

## Abbreviations

NET: neuroendocrine tumor
CNV: copy number variations
dNET: duodenal NET
FFPE: formalin-fixed paraffin embedded
HCTS: high-coverage targeted sequencing
iNET: ileal NET
IHC: immunohistochemistry
NET: jejunal NET
LOH: loss of heterozygosity
TMA: tissue micro-array
WHO: World Health Organization
