# Supplementary figures and images for: Mutational and copy number asset of primary sporadic neuroendocrine tumors of the small intestine
Source: Virchows Arch. 2018 Sep 16;473(6):709–17. doi: 10.1007/s00428-018-2450-x (PMC6267237; doi:10.1007/s00428-018-2450-x)

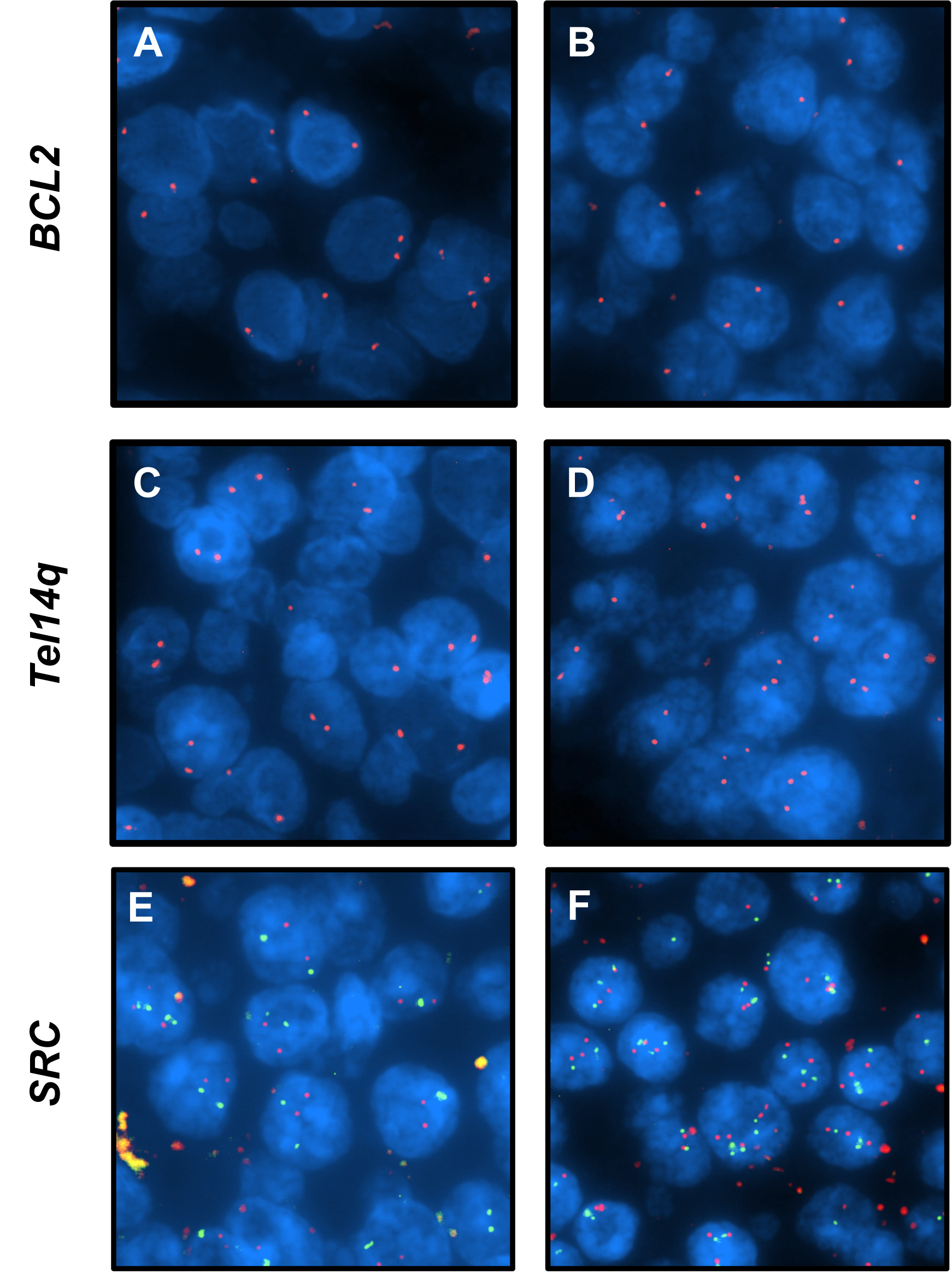

Supplement: Supplementary file 1 — FISH images of representative CNV alterations in SI-NET samples. A) Diploid BCL2 status; B) Monosomy of BCL2; C) Diploid Tel14q status; D) Polysomy of Tel14q; E) Diploid SRC status; F) Polysomy of SRC. (PNG 2047 kb) [file 428_2018_2450_Fig3_ESM.png]

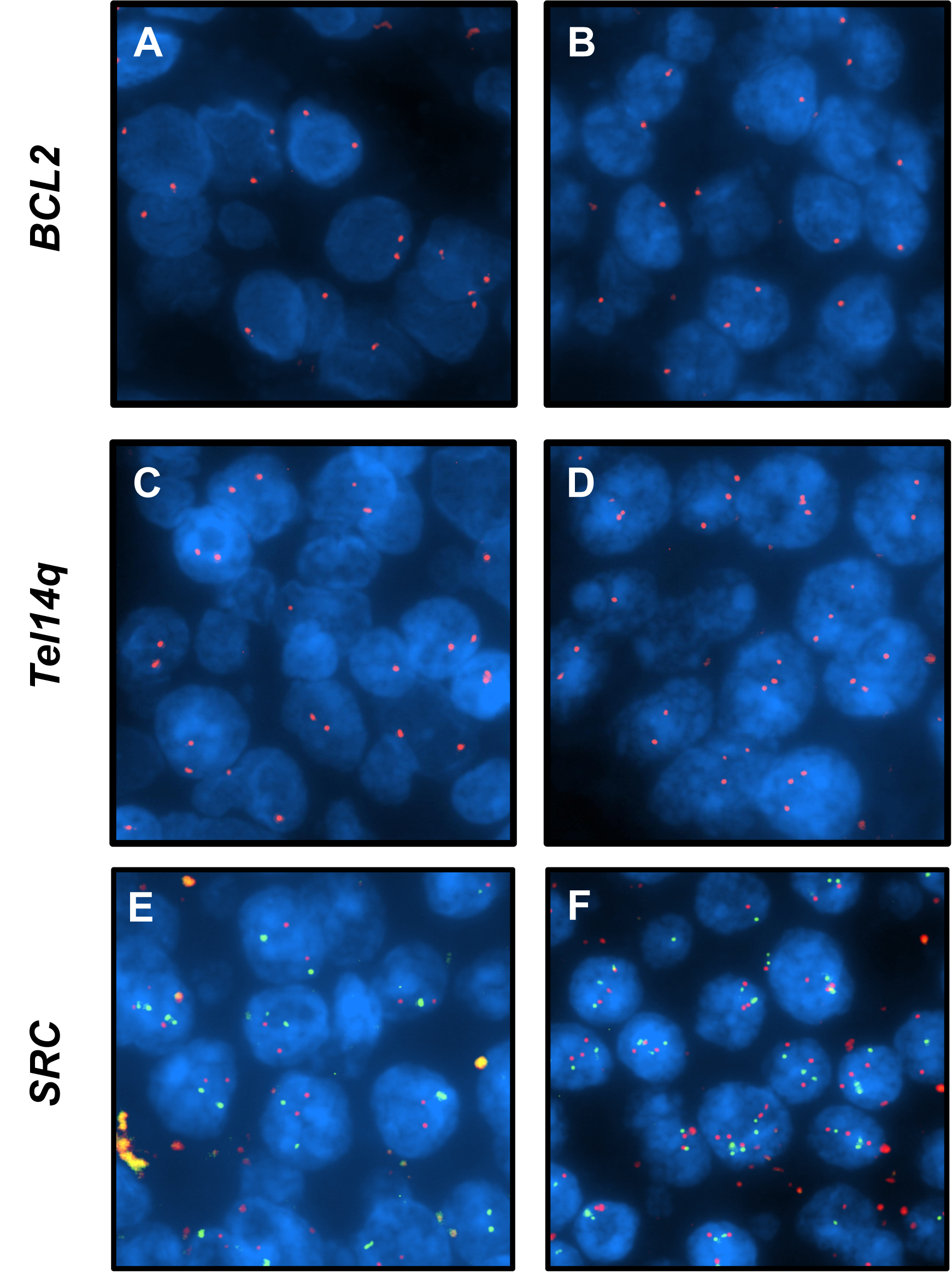

Supplement: Supplementary file 2 — High Resolution Image (TIF 11912 kb) [file 428_2018_2450_MOESM1_ESM.tif]

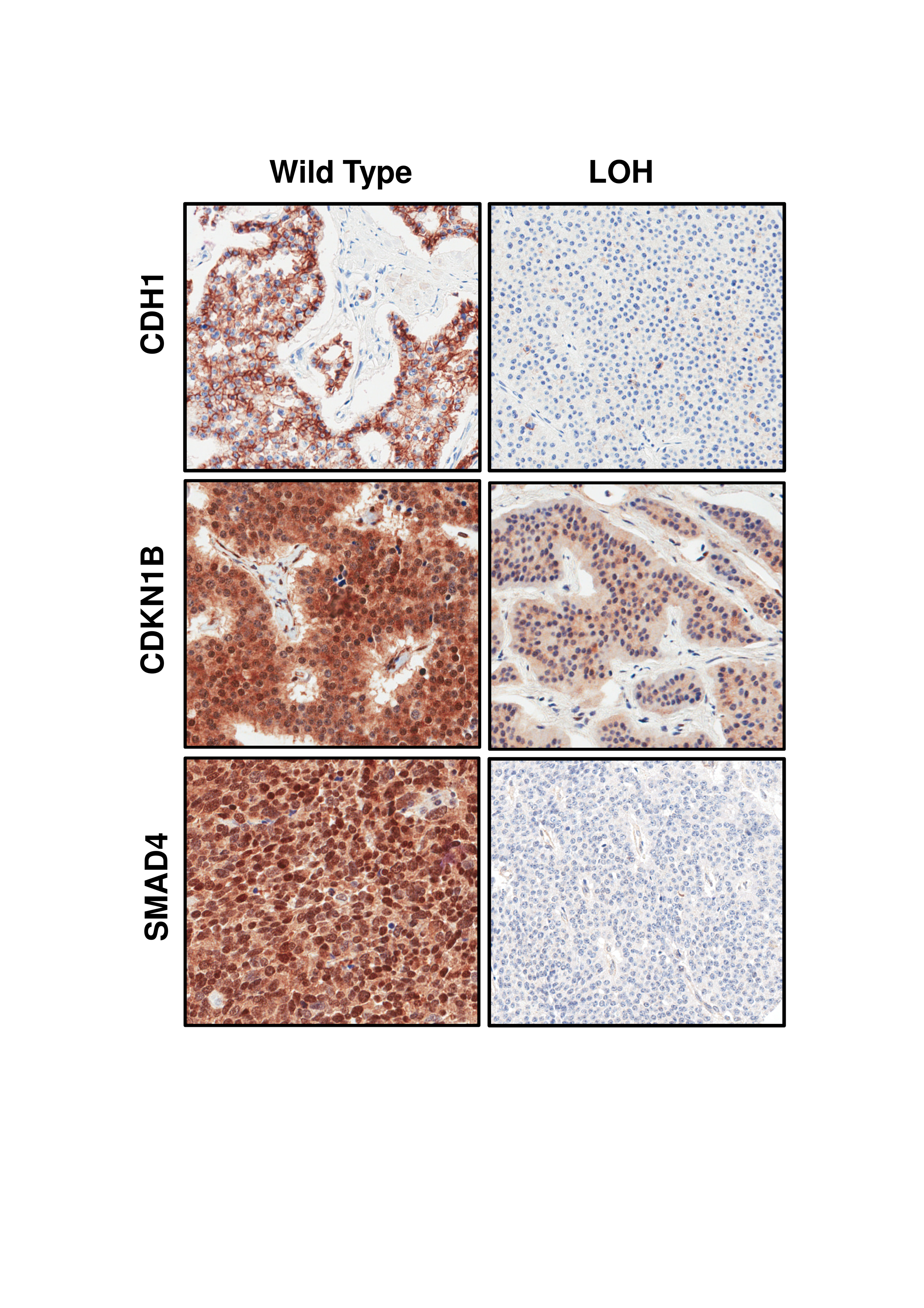

Supplement: Supplementary file 3 — Immunohistochemical validation of the expression of E-cadherin (CDH1), p27 (CDKN1B) and smad4 (SMAD4) proteins in mutated and wild type tumors. Representative immunohistochemical staining of the products of the CDH1 (E-cadherin), CDKN1B (p27) and SMAD4 (smad4) genes in wild type tumors (left panels) and tumors characterized by LOH of the corresponding genes (right panels). The wild type tumors retained a normal protein expression, which was lost in LOH cases. (PNG 5738 kb) [file 428_2018_2450_Fig4_ESM.png]

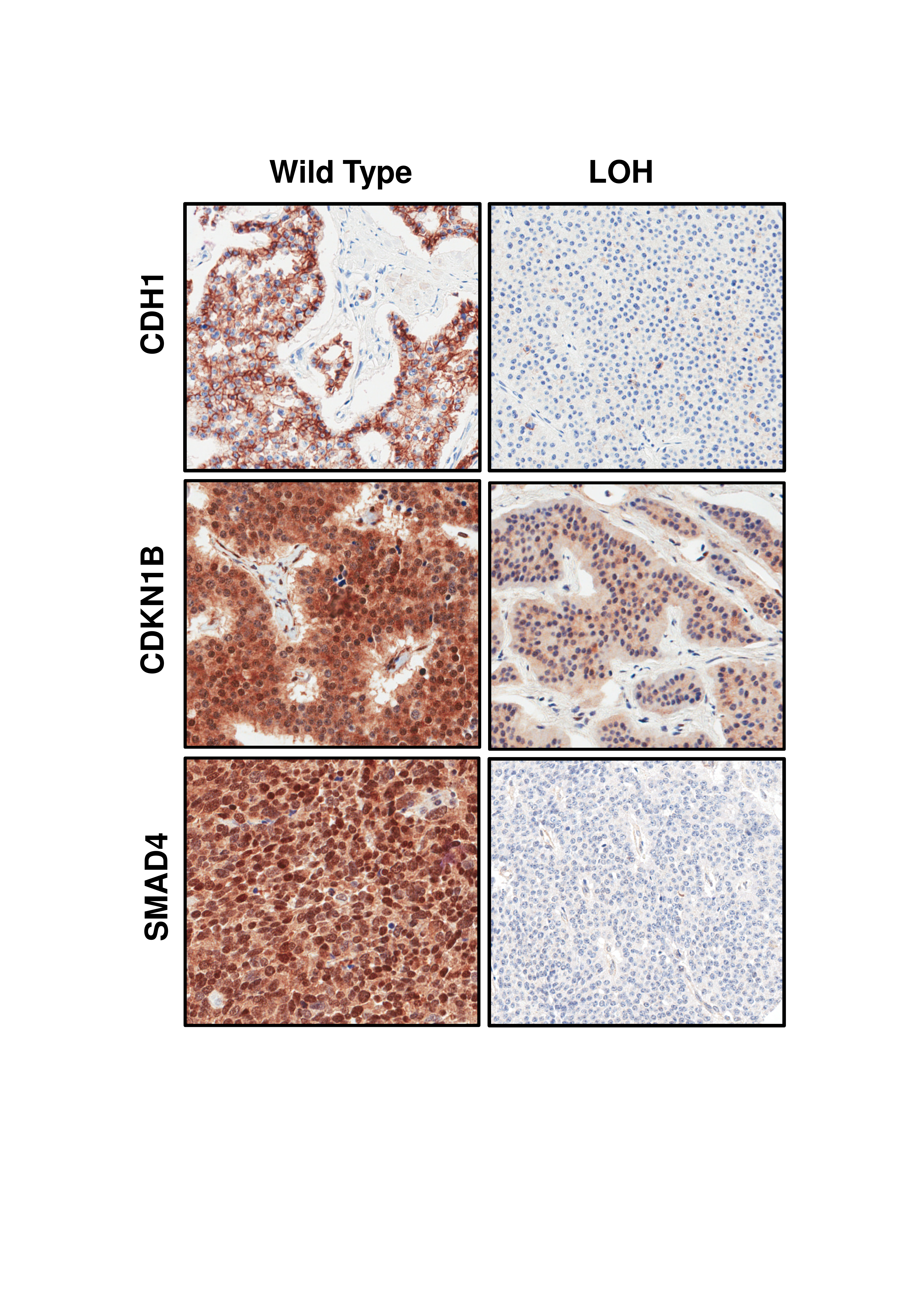

Supplement: Supplementary file 4 — High Resolution Image (TIF 24929 kb) [file 428_2018_2450_MOESM2_ESM.tif]

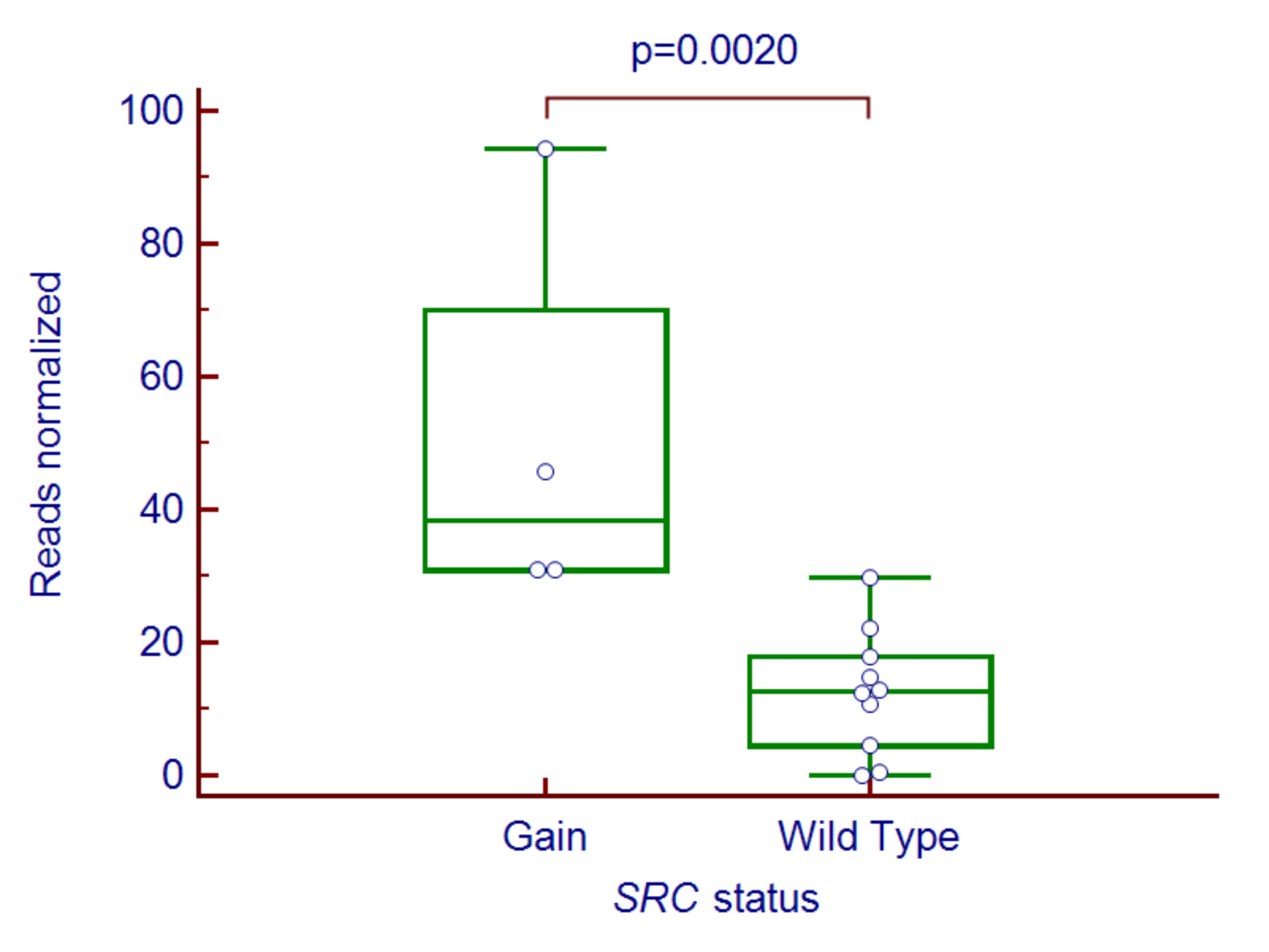

Supplement: Supplementary file 5 — Small intestinal neuroendocrine tumors with copy gain of SRC locus display enhanced levels of SRC mRNA. Normalized expression reads of 14 samples grouped according SRC copy number status in Gain (4 samples) and Wild-Type (10 samples). (PNG 104 kb) [file 428_2018_2450_Fig5_ESM.png]

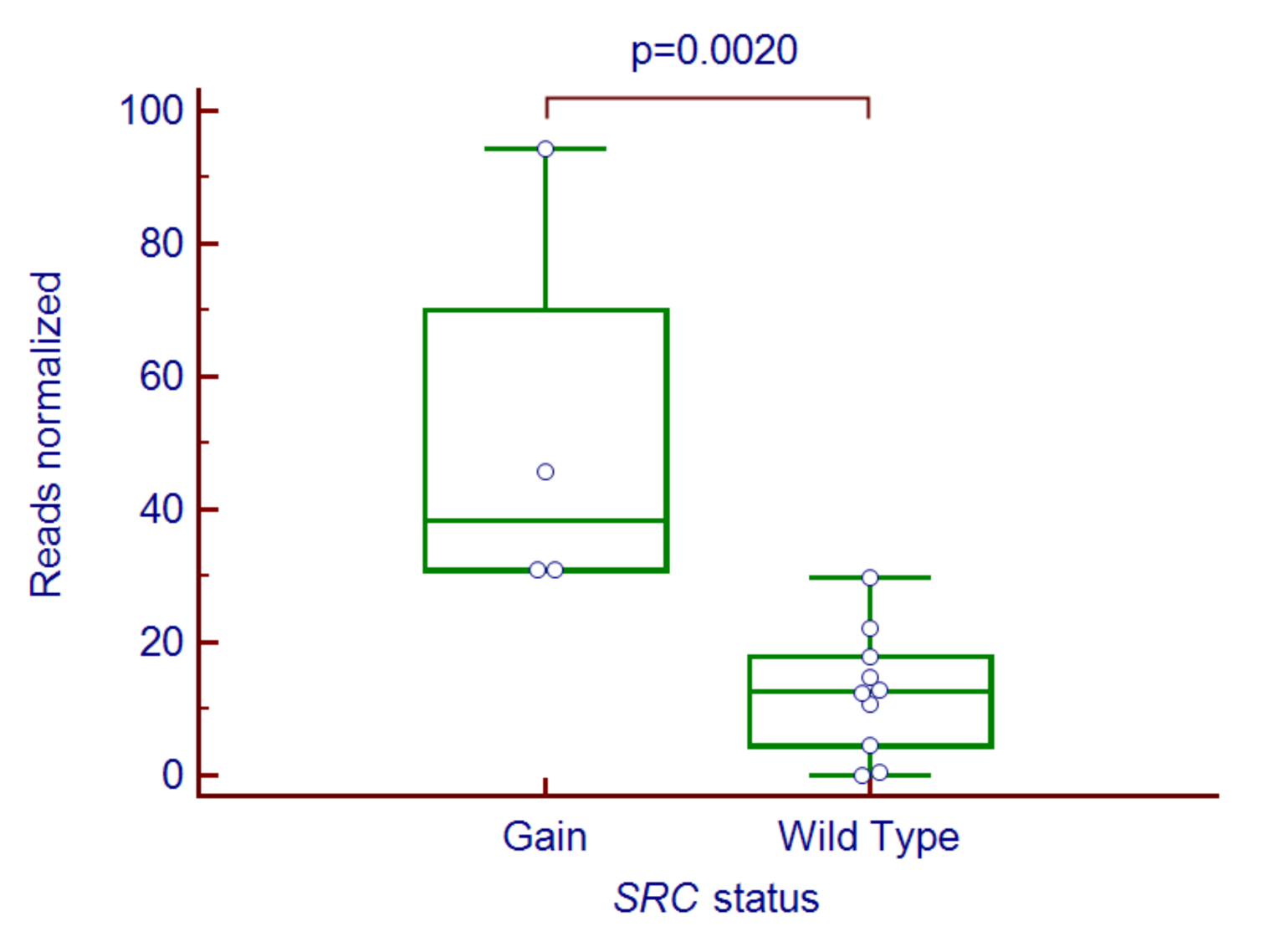

Supplement: Supplementary file 6 — High Resolution Image (TIF 5078 kb) [file 428_2018_2450_MOESM3_ESM.tif]
